# Effects of Silicon Amendment on Soilborne and Fruit Diseases of Avocado

**DOI:** 10.3390/plants6040051

**Published:** 2017-10-20

**Authors:** Elizabeth K. Dann, Duy P. Le

**Affiliations:** Queensland Alliance for Agriculture and Food Innovation, University of Queensland, Brisbane, QLD 4072, Australia

**Keywords:** Phytophthora, potassium silicate, available silicon, anthracnose, stem end rot

## Abstract

The effects of silicon (Si) amendment have been studied in several plant/pathogen interactions; however, studies in horticultural tree crops are limited. Effects of amendment with soluble potassium silicate (AgSil^®^32, approximately 30% available Si), or milled cement building board by-products (Mineral Mulch (MM) or Mineral Dust (MD), containing 5% available Si) were investigated in field and greenhouse trials with avocado. Orchard soil drench applications with potassium silicate improved yield and quality of fruit, but visual health of trees declining from Phytophthora root rot (PRR) was not affected. Orchard spray or trunk injection applications with potassium silicate were ineffective. Amendment of potting mix with MM and MD reduced root necrosis of avocado seedlings after inoculation with *Calonectria ilicicola*, an aggressive soilborne pathogen causing black root rot. Application of MM to mature orchard trees declining with PRR had a beneficial effect on visual tree health, and Si accumulation in leaves and fruit peel, after only 10 months. Products that deliver available Si consistently for uptake are likely to be most successful in perennial tree crops.

## 1. Introduction

Silicon (Si) has been shown to reduce disease and increase plant growth in a number of agricultural crops, predominantly cereal grains and dicotyledonous annual crops such as cucumber and tomato, and the topic has been recently reviewed [1,2]. Likely mechanisms include formation of physical barriers to pathogen ingress, activation of biochemical plant defenses, and influence on absorption of essential nutrients. The benefits of Si amendment for ameliorating toxicities and abiotic stresses is also well established [3], which may also influence plant health and disease suppression, although those interactions have not been thoroughly investigated. The reports concerning effects of amendment with soluble Si to horticultural tree crops is still scarce. Field or soil applications of various formulations and concentrations of Si to mango, citrus or apple reduce diseases caused by bacterial [4,5] or fungal pathogens [6,7,8,9]; enhance resistance to insect pests [10] and physiological disorders [11]; and increase yield [7], fruit size [12], and biomass [13].

Reports of Si amendment in avocado primarily concern effects on fruit quality including postharvest physiology and anthracnose disease, caused by *Colletotrichum* spp. and health of trees affected by Phytophthora root rot (PRR), caused by *Phytophthora cinnamomi* (Pc). Many of these reports have not been published in the mainstream literature, thus are reviewed in detail below.

Studies undertaken in 2004 provided the first indication that soluble Si, applied as trunk injections (common in avocado for the delivery of phosphonate to protect roots from PRR), could reduce anthracnose development as fruit ripen after harvest. “Hass” avocado trees carrying crops of developing fruit were injected with approximately 60 mL of 1000 ppm potassium silicate, and fruit harvested for quality assessments 2, 8 and 12 weeks later. There was no significant difference in anthracnose disease when harvested two weeks after injection treatment, however, anthracnose was significantly reduced by Si injection compared with controls when fruit were harvested and ripened at the later harvests, with 80% reduction in severity, and increased shelf life, recorded in fruit harvested two months after Si injection treatment [14]. A subsequent study confirmed that injecting with 1000 or 2000 ppm potassium silicate during early fruit development (28 weeks prior to harvest), or 12 weeks prior to harvest, reduced anthracnose development compared with untreated controls by at least 40%. Treatment with 1000 ppm during fruit development and 2000 ppm 12 weeks prior to harvest had greater than 60% reduction in fruit anthracnose which was significantly less than that recorded in fruit from untreated trees. Interestingly, 1000 ppm injected at each of the treatment times did not significantly reduce anthracnose, and there was no effect on stem end rot disease in fruit [15]. Five orchard sprays at intervals of approximately six weeks throughout fruit development with 1000 ppm potassium silicate were not effective at reducing postharvest disease or improving marketability of “Hass” avocado fruit, compared with industry standard fungicide applications [16].

The effects of Si soil drench applications in orchards, or as post-harvest applications, on avocado fruit physiology and accumulation of Si have been assessed. Soil drench applications (concentrations not reported) in-field during fruit development had no effect on accumulation of Si in the exocarp and mesocarp of fruit, or on CO_2_ production in ripe fruit [17]. However, dipping harvested fruit into high concentrations (<2500 ppm) of potassium silicate suppressed respiration (reduced CO_2_) and ethylene production. Energy dispersive X-ray analyses demonstrated that Si accumulated in the exocarp of such fruit approximately 14% wt, with <1% in the mesocarp. Although no data were presented, the authors stated that none of the Si treatments affected anthracnose disease in fruit, and thus concluded that, despite the suppression of ripening parameters, an enhanced shelf life was not achieved because of the severity of postharvest disease [17]. Similarly, there was no significant difference in anthracnose upon ripening when harvested fruit were dipped in 1000 ppm potassium silicate, compared with untreated control fruit, although there was an indication of enhanced defense enzyme activity [18]. Tesfay et al. [19] reported the potential of potassium silicate postharvest treatment to improve avocado fruit quality via maintenance of fruit moisture, increased catalase enzyme activity, and increased accumulation of phenolic compounds.

In preliminary greenhouse tests with one-month-old avocado seedlings, several Si products, including potassium silicate), calcium silicate (wollastonite) and magnesium silicate were not as effective at reducing root necrosis caused by Pc as phosphonate, a standard treatment for PRR [20]. There were some indications of root health improvement following some treatments, but effects were inconsistent. Conversely, greenhouse tests undertaken in South Africa with advanced nursery stock (12–18 months old) showed that one soil drench application of potassium silicate before and four applications after inoculation with Pc resulted in significantly healthier root systems than inoculated control plants, and were comparable to those treated with phosphonate, in two of four experiments [21]. Root mass and new root growth was increased by Si, suggesting that Si may stimulate root growth and fortification under high Pc disease pressure. A single Si soil drench application a day prior to inoculation was not effective at reducing root necrosis, and the authors suggested that for effective disease suppression, plant-available Si should be applied continuously, due to the rapid polymerization of soluble Si. A day was most likely not sufficient time for Si to be translocated and deposited and for defenses to pathogen infection to be activated.

Effects of soil drench applications on canopy health and root densities were assessed in an orchard trial with mature trees. Twenty liters per tree of potassium silicate, 20 mL/L (approximately 4000 ppm), was applied once, twice or three times at four monthly intervals, in the first year, and repeated in Year 2. Two or three applications resulted in significantly healthier canopies, compared with untreated trees, which declined in health over time [22,23]. The trees receiving two or three Si treatments per year had the highest root densities (determined by image analyses of 0.5 m^2^ soil surface area of each tree), compared with controls or those receiving industry standard injections with phosphonate, suggesting that Si may aid in the regeneration of roots in PRR-compromised trees. One or two soil drench applications with Si reduced tree yields, however, three applications increased yields. The authors suggested that the optimal timing of applications was during flowering/fruit set, before fruit drop, and prior to the summer root flush, however the timing of applications should be determined by soil structure as Si leaches easily, i.e., sandy soil will require more regular applications. There were inconsistent effects against postharvest diseases (anthracnose and stem end rot) and physiological disorders of fruit. Stem injections of Si did not improve tree health or increase root growth [23]. There was an indication of increased leaf boron uptake in Si treated trees, but no consistent effect on leaf or root Si content [23]. Dixon et al. [24] applied sodium metasilicate as a soil drench (delivering 100 or 200 ppm a.i. soluble Si), to avocado trees in severe decline due to PRR, and assessed tree health 6 and 13 months later. The single application did not significantly improve tree health at the final assessment; however, there was a slight improvement in trees treated with the higher rate. Root health was improved at six months compared with untreated controls, and the authors concluded that multiple applications may have been more effective.

Phytophthora root rot remains the key challenge to orchard productivity for avocado growers in Australia, particularly in the eastern tropical and sub-tropical regions which have encountered high rainfall in recent years, favoring this root disease. PRR has significantly impacted productivity at orchard and whole-industry level due to tree decline and death, an abundance of small fruit which can be difficult to market, and poorer quality fruit from PRR affected trees [25]. The nectriaceous soilborne fungi *Calonectria ilicicola* and *Dactylonectria* spp. are common nursery pathogens infecting roots and may cause tree deaths with 12 months of planting [26,27]. The most problematic fruit diseases are anthracnose, caused by *Colletotrichum* spp. and stem end rots, caused by *Colletotrichum* spp., and/or fungi in the family Botryosphaeriaceae [25]. Currently, orchard management includes a range of cultural and chemical practices, such as optimized drainage, irrigation and fertilizer regimes, mulching and targeted sprays with fungicides for fruit disease and injections with phosphonate for PRR. It is wise for the industry to remain open-minded about alternative or complementary management options. The successful management of pests and diseases will rely on an integrated approach, which utilizes as many cultural, chemical and agronomic options as possible.

The aim of the current research was to investigate effects of two products with high available Si contents, for their effects on avocado canopy health, fruit quality and accumulation of Si. Greenhouse trials were undertaken over 3–4 months where treated seedlings were inoculated with root pathogens *Calonectria ilicicola* or *Phytophthora cinnamomi*. Trials at several sites were conducted over 2–3 years in established orchards where trees were declining due to PRR. Potassium silicate (SiO_2_/K_2_O), for example AgSil^®^32 (PQ Australia P/L), has been available for many years, and has been used in many of the studies described above. Mineral Mulch (MM) and Mineral Dust (MD) are cement building board by-products available commercially in Australia from Shiftwaste P/L since 2014. Research with MM to date has concentrated on sugarcane, a Si accumulator, where yield increases of up to 16% above untreated controls have been recorded after its application [28]. Anecdotal data also showed positive response of capsicum and macadamia to MM; however, neither MM nor MD have been evaluated in avocado. Trials in commercial orchards over multiple years are essential, where assessments of yield, tree health, fruit quality and packout data are critical for determining the role of such amendments in improving orchard productivity.

## 2. Results

### 2.1. Field Trials with AgSil 32 (2014–2016)

Trials were conducted on-farm at Childers, Goodwood, Beechmont (QLD) and Comboyne (NSW). At Site 1, Childers, there were no significant effects on tree health of Agsil drench applications (5000 ppm solution of Si delivered in 20 L water per tree on three occasions annually) eight months to two years after the initiation of the trial (Table 1). In April 2015, one year after commencement of the trial, images of root growth within the “windows” placed under each tree were captured, and analyzed with WinRhizo software. There were no significant differences in root lengths, area or diameter between the trees treated with Agsil drench vs. non-drenched trees (data not shown). Yields were recorded mid-way through the trial in July 2015 when yields from Agsil drenched trees were not significantly different compared with non-drenched trees (Table 1). Single bins of fruit from each treatment were run through a commercial packingshed (hence no statistical analysis was possible). There were small increases in the percentage of fruit in premium grade and total percent packouts from the Agsil drench treated trees, however, there was a 40% increase in 2nd grade fruit (and decrease in 3rd grade fruit) from Agsil-drenched trees compared with non-drenched trees (Table 2).

The trial at Site 2, Goodwood, (QLD), consisted of two rows of trees treated with Agsil drench (5000 ppm Si per tree delivered in 20 L water) in two separate blocks, with untreated rows either side assessed as the untreated controls. Agsil did not consistently improve tree health compared with untreated trees at any time point, however tree yields from Agsil drenched trees were approximately 13% and 39% higher than trees in the untreated rows for the two blocks (data not presented).

Treatments to trees at Site 3, Beechmont, (QLD), were undertaken by project staff, (not the orchard operator), and included Agsil soil drenches (5000 ppm solution of Si delivered in 20 L water per tree), foliar spray (3300 ppm solution of Si delivered in 5 L water per tree) and trunk injection (3000 ppm solution of Si delivered in 5 × 20 mL injection sites per tree). Tree health was not significantly affected by Agsil treatment at any of the assessment dates between May 2014 and August 2016 (Table 3). A tray of fruit from each trial tree was harvested at commercial maturity in 2015 and 2016 and assessed for postharvest disease. There were no significant differences in severity or incidence of anthracnose among treatments in either 2015 or 2016, however fruit from trees sprayed or drenched with Agsil had significantly less severe stem end rot than those that were injected in 2015 (Table 4). There were no significant effects of Agsil on stem end rot in 2016 (Table 5).

Agsil was applied six times to selected trees in the trial at Site 4, Comboyne, (NSW), between August 2014 and June 2016. Health of Agsil drenched trees had improved by the final assessment compared with untreated controls, but the effect was not statistically significant (Table 6). However, estimated yield was 14% higher for the Agsil drenched trees (Table 6). One bin of fruit from each treatment was transported to a commercial packingshed to obtain packout data. While the percentage of fruit in the Premium grade was similar for fruit from the two treatments, there were more 2nd grade from Agsil treated trees than from untreated controls (Table 7).

### 2.2. Greenhouse Trials with Mineral Mulch and Mineral Dust 

#### 2.2.1. Experiment 1

**Figure 1:**
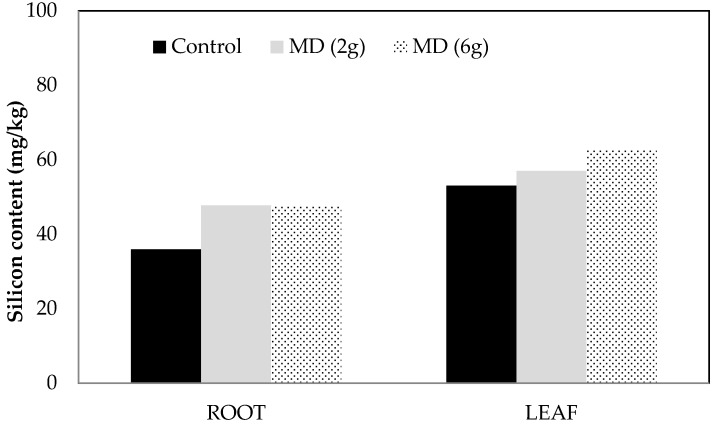
Silicon content (mg/kg) in dried root and leaf tissue four weeks after treatment with Mineral dust (MD), Experiment 1. Only plants from uninoculated treatments were analyzed. Control plants received no MD; MD (2 g), plants received 2 g MD in total; MD (6 g), plants received 6 g MD in total. Root and leaf, No significant differences among treatments (*p* > 0.05, *n* = 6).

Twenty-eight days after inoculation with *C. ilicicola* (Ci), seedlings were significantly shorter and had reduced leaf/stem and root biomass, and a greater proportion of necrotic roots, compared with those which were uninoculated (Table 8). There was no significant effect of MD treatment on plant height, compared with plants which did not receive MD amendment. There were indications of a MD effect amongst the uninoculated treatments, where the healthiest roots (least necrosis) were from seedlings receiving 2 g MD, and were significantly healthier than those receiving 6 g MD (Table 8). Si concentration in leaves and roots of seedlings treated with 2 g and 6 g MD were not significantly different from those receiving no MD (Figure 1).

#### 2.2.2. Experiment 2

**Figure 2:**
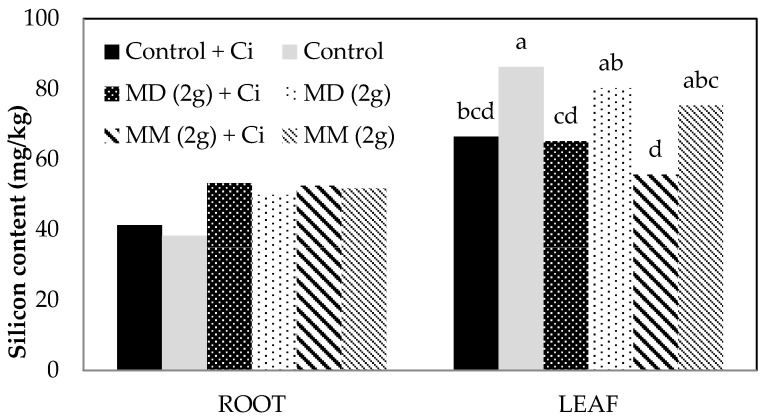
Silicon content (mg/kg) in dried root and leaf tissue four weeks after treatment with Mineral dust (MD) and Mineral mulch (MM), Experiment 2. Control plants received neither MD nor MM; MD (2 g), plants received 2 g MD in total; MM (2 g), plants received 2 g MM in total; control + Ci, MD (2 g) + Ci, MM (2 g) + Ci, plants inoculated with Ci; Root, No significant differences among treatments, (*p* > 0.05, *n* = 6); Leaf, Bars labeled with the same letter are not significantly different (*p* < 0.05, *n* = 6).

The treatments for the second experiment were modified to replace 6 g MD treatment with 2 g MM. There was a significant effect of Ci reducing plant height over the four-week duration of the experiment, although not as severely as in Experiment 1. There were no significant differences in seedling growth among treatments when comparing respective inoculated and uninoculated seedlings (Table 9). Leaf and stem and root biomass (FW and DW) were not significantly reduced by fungal inoculation within treatment comparisons (Table 9). Both MM and MD significantly reduced the percentage of necrotic roots compared with non-amended seedlings when assessed four weeks after inoculation (Table 9). Inoculation with Ci reduced Si accumulation in roots compared with respective non-inoculated plants, however, there were no significant differences in Si accumulation in leaves or roots of MM or MD treated seedlings compared with respective Ci-inoculated or uninoculated controls (Figure 2).

#### 2.2.3. Experiment 3

*Phytophthora cinnamomi*, the oomycete pathogen causing PRR, was included as a second inoculation treatment in Experiment 3. There were significant differences amongst treatments for all measured parameters. Inoculation with either Ci or Pc significantly reduced seedling growth, root biomass and increased severity of root rot (Table 10), with effects of Pc inoculation generally (but not always statistically) worse than Ci. Amendment with either MD or MM did not affect plant growth (height), biomass or root necrosis compared with respective uninoculated or inoculated controls. Root necrosis was approximately 16% or 24% less severe in plants treated with MD or MM, respectively, before inoculation with Ci compared with untreated controls, however the difference was not statistically significant (Table 10). There were no leaf and root nutrient analyses obtained for this experiment.

### 2.3. Preliminary Field Trials with Mineral Mulch

Table 11 shows the average tree (canopy) health for treated and untreated trees at each site at the date of MM application (July 2016) and at the first assessment (May 2017). At the Kumbia site, all trees improved in health, irrespective of treatment, and, in May 2017, there was no significant difference in canopy health between treatments (Table 11). At Kumbia, there were no differences in heights and diameters between trees treated with MM or those which received no treatments (data not shown).

At the Goodwood orchard, canopy health in 2016 was significantly worse in the trees selected for MM application than those in adjacent rows selected as the untreated controls (Table 11). However, 10 months after MM application, the tree health had improved considerably, to be the same as that in untreated trees. A similar trend was also observed in the Childers orchard, where the improvement in tree health over time was more rapid for the MM treated trees than untreated controls (Table 11).

Fruit were collected in 2017 for postharvest disease assessments and nutrient analyses. There were no differences amongst orchards or treatments in the interval between harvest and “eating ripe” stage. There were significant differences among treatments (and orchards) in severity of anthracnose disease, where fruit from Kumbia or Childers treated with MM, or Childers untreated, had significantly more disease than from Goodwood, or Kumbia untreated (Table 11). A similar trend was observed in stem end rot. These results must be viewed with extreme caution, as most trees from Kumbia and Childers had no fruit, so a composite sample from only a few trees was available for testing. There were also limited numbers (replicates) from each treatment at each site. The usual practice for fruit assessments is to harvest a full tray of fruit (18–20 pieces) from a replicate tree, ensuring at least six replicate trees per treatment. Future trials should include more treated trees and incorporate more robust sampling and replication.

**Figure 3:**
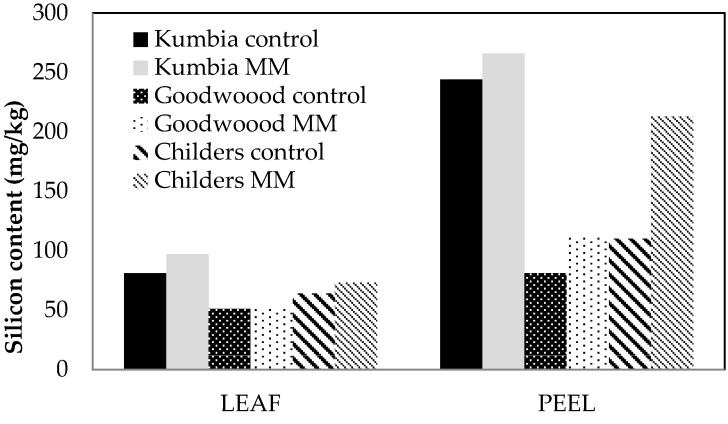
Silicon content (mg/kg) in dried avocado leaf and peel tissue 10 months after treatment of avocado trees at three locations in Queensland with Mineral Mulch (MM). *n* = 1 and thus no statistical analysis was undertaken.

Si concentration in leaves and fruit peel was determined, however, it must be noted that statistical analyses were not performed on the data, as there was only one replicate sample for each tissue type and treatment at each site. Si concentration in leaves was higher in MM treated trees at two sites, Kumbia and Childers, and higher in fruit peel at each site, compared with leaves and peel from untreated control trees (Figure 3). This provides strong indication that Si is taken up by roots in mature orchard trees and translocated and deposited in leaves and fruit peel, within a relatively short time frame (10 months).

## 3. Discussion

The four trials with soluble potassium silicate (AgSil^®^32) conducted on growers’ orchards highlight the inconsistent effects of Agsil under field conditions. Tree health was generally improved in Agsil treated trees, but not significantly compared with untreated trees. In two of the three trials where yields were measured, average yields per tree were 13–39% greater from Agsil-drenched trees compared with untreated trees. Fruit quality and packout data demonstrate that, after two years of application, fruit from Agsil treated trees had higher proportions of fruit in 2nd grades (but not Premium grade), superior quality with reduced stem end rot and defects attributed to pepper spot (superficial necrotic lesions <0.5 mm, caused by *Colletotrichum* spp.), than fruit from non-drenched trees. One packingshed estimated the improved quality represented a 20% increase in net return to the grower, based on prices received at the time of packing.

Fruit postharvest evaluations from one trial showed that stem end rot was significantly reduced in the first year of Agsil treatment, resulting in a greater proportion of marketable fruit. Anthracnose was not reduced by potassium silicate drench treatments. Previous studies showed the reverse with potassium silicate injection treatments, i.e., reducing anthracnose but having no effect on stem end rot [14,15]. Lack of effect of spray applications on disease severity was, however, consistent with a previous study [16]. It is possible that more frequent applications of Agsil targeted more closely to autumn and spring root flushes, may improve uptake and translocation of available Si and hence efficacy.

While the disease-reducing beneficial effects of Si applications are most frequently attributed to enhancement of physical barriers to pathogen ingress and activation of plant defense mechanisms, a direct fungicidal effect is also possible, depending of course on the formulation of Si and its applied concentration. Soluble potassium silicate (20.7% SiO_2_) was tested for its effects on *in vitro* growth of several phytopathogenic fungi including *Sclerotinia sclerotiorum*, *Fusarium oxysporum*, *F. solani*, *Colletotrichum coccodes*, *A. solani* and oomycetes, *P. cinnamomi* and *Pythium* sp. [30]. Concentrations of 5 mL and 10 mL potassium silicate/L agar completely inhibited growth of *P. cinnamomi* and *S. sclerotiorum*, however, mycelial growth of *F. oxysporum*, *F. solani* and *Verticillium theobromae* was increased compared with controls at these concentrations. Growth was inhibited at the higher concentrations tested, 20, 40 and 80 mL/L agar [30]. Potassium silicate amendment increased pH of media, however, mycelial growth of all fungi tested occurred at corresponding high pH (adjusted with KOH), demonstrating the fungicidal effect of the potassium silicate solution was not simply due to alkalization of the agar media [30]. This information is important when considering field applications. It would be counterproductive to apply a treatment which enhanced growth of an economically important pathogen rather than suppressing it! An effect of Si on pathogen or other microbial populations (including beneficial or suppressive microbes) and diversity in soil has not been reported, but requires further investigation.

There are strong indications that Mineral Mulch and/or Mineral Dust amendment to potting mix of greenhouse avocado plants reduces the root necrosis caused by *C. ilicicola*, the nectriaceous fungus causing black root rot of avocado.

In greenhouse Experiment 3, it is clear that the inoculation with *P. cinnamomi* and subsequent conditions favouring disease were too severe, and there was little chance of observing treatment effects when seedlings were overwhelmed by the pathogen. In a fourth greenhouse experiment, there was an indication that treatment with MD and MM reduced root necrosis after inoculation with either Ci or Pc (data not shown). MD reduced necrosis by approximately 30% and 18%, while MM reduced necrosis by approximately 19% and 9%, compared with Ci or Pc inoculated controls, respectively. This was also observed for MD and MM in Ci inoculated plants, in Experiments 2 and 3, where MD appeared to be more effective at reducing necrosis than MM. However, the higher rate, 6 g, of MD applied in greenhouse Experiment 1, was likely to have been too high, and resulted in less healthy (lower biomass and more necrosis) than those treated with 2 g. Perhaps 6 g MD was too much in a short timeframe and overwhelmed plant defense responses. It is possible that the smaller particle size of MD contributed to faster nutrient uptake and defense responses than MM, but this requires further investigation, and supports the idea of a combined MD + MM treatment compared with MD and MM alone, in planned field trial investigations.

Si accumulation in roots and leaves were measured in dried tissue from two greenhouse experiments. Amendment with MD or MM enhanced accumulation by around 30% in roots compared with untreated controls (Figure 1 and Figure 2). However, smaller increases (7–18%) in leaf Si accumulation were observed in Experiment 1, while concentration actually decreased compared with untreated controls in Experiment 2.

In preliminary field trials with MM, application at approximately 2 t/Ha had a beneficial effect on tree health at two sites, and Si accumulation in leaves and fruit peel, after only 10 months. Some larger particles of MM were still clearly visible underneath trees at each site, and may provide a consistent and sustained delivery of Si over a period of time. These preliminary results are extremely promising and warrant further replicated field trials, with suggested treatments of MM at 2 and 4 t/Ha and a combined MM and MD application. MD may provide a rapid delivery of Si, while the larger particle size of MM ensures release over several months. Canopy health as well as fruit yields and quality, and nutrient analyses will be necessary to evaluate the efficacy and cost benefit of MM applications in commercial avocado orchards. Comprehensive soil testing, including microbial and chemical (nutrient, pH, EC) analyses would also be desirable. Future trials should evaluate treatments applied to whole rows (e.g., 30 trees), compared with grower standard treatment, at different orchards across Australia (for replication), and across at least three years. Whole-row treatments can be assessed for productivity (yield) as well as commercial packout and quality. This approach would give more meaningful and realistic outputs.

## 4. Materials and Methods

### 4.1. Field Trials with AgSil32

AgSil^®^32 is a soluble potassium silicate product from PQ Australia Pty. Ltd., and contains 32% *w*/*v* SiO_2_ and 21% *w*/*v* K_2_O.

Field trial sites were conducted at 4 locations between Central Queensland and the mid New South Wales coast to assess the effect of potassium silicate on tree health and productivity by reducing PRR. Each site was visited between April and June 2014 to establish trial sites, establish baseline tree health and establish communication links with growers for potassium silicate application and ongoing management. Grower collaborators were responsible for Agsil applications at three sites (all drench applications), and the pathology team independently conducted the 4th trial on an orchard at Beechmont, QLD. The selected trial sites enabled evaluation of the potential for potassium silicate application to manage PRR across 3 growing regions, with unique orchard management activities at each site.

Agsil was applied as a drench at 300 mL/tree delivered in 20 L (5000 ppm solution of Si delivered in 20 L water per tree), to the active root zone. Application was planned to coincide with periods of active root flush (autumn and summer), however, frequency and timing varied across sites for a number of reasons. Tree canopy health was determined at each site visit by visual assessment according to a widely-used rating scale [29]. Fruit yields were collected from Sites 1 and 2 in 2015, and Site 4 in 2016. Additionally, commercial packout data were obtained for fruit from Site 1 in 2015 and Site 4 in 2016. Fruit was harvested from trees in the fully replicated trial at Site 3 in 2015 and 2016, ripened and assessed for development of postharvest anthracnose and stem end rot.

Site 1 (Childers, QLD): The entire orchard is currently treated with a low dose of potassium silicate on a monthly basis by fertigation. Sick/declining trees were treated at a high “treatment” rate of 300 mL Agsil per tree (5000 ppm solution of Si delivered in 20 L water per tree) on 3 occasions annually. Given the entire orchard currently receives Agsil, this trial was set up to examine the effects of withdrawing Agsil treatment from selected trees with obvious signs of decline due to PRR. A total of 22 trees were selected for monitoring over the duration of the trial, with 11 of these clearly identified for Agsil treatment withdrawal. At the trial initiation, the average health ratings of trees subject to Agsil withdrawal and continuing treatment was 5.36 and 5.67 respectively. The tree health rating system used across all trial sites is based on the established system, where 1 = totally healthy and 10 = dead tree [29]. Root windows to facilitate measurements of root growth were installed under a selection of Agsil drench and non-drenched trees. Trees received 300 mL Agsil per tree, delivered in 20 L of water at each treatment date throughout the trial (February, May, July and October 2014; February, May, August and December 2015; and May 2016).

Site 2 (Goodwood, QLD): This trial site was established on a well-managed orchard with no history of potassium silicate treatment. The trial consisted of two treatment rows in which potassium silicate soil drenches were applied, one in a block of relatively “healthy” trees, and the other row within a block of “sick” declining trees. Rows either side of the Agsil treated rows were assessed for canopy health as the untreated controls. Trees received 300 mL Agsil per tree, delivered in 20 L of water (i.e., approximately 5000 ppm) at each treatment date throughout the trial (July, October and December 2014; October and November 2015; and March 2016). Yield data was collected in May 2015.

Site 3 (Beechmont, QLD): This trial site was established on a small orchard with no history of potassium silicate treatment. Trees currently receive a phosphorous acid injection annually, however an area of the orchard in mid-decline was selected to evaluate a range of potassium silicate application methods. Treatments were undertaken by project staff, (i.e., not the grower), and included AgSil soil drenches as per trial sites 1 and 2 (300 mL Agsil per tree, delivered in 20 L of water, i.e., 5000 ppm solution), foliar spray (50 mL Agsil per tree delivered in 5 L water, i.e., 3300 ppm solution) and trunk injection (approximately 1 mL Agsil per tree in 100 mL water, i.e., 3000 ppm solution delivered in 5 × 20 mL injection into vascular tissue per tree). In 2014 trees were treated in June (all treatments) and December (spray and drench), 2015 trees treated in May (all treatments) and December (spray and drench), 2016 treated in May (spray and drench) and June (all treatments). Tree health assessments have been obtained at least twice each year, and fruit were harvested for quality assessments in 2015 and 2016.

Site 4 (Comboyne, NSW): This trial site was established on an orchard with no history of potassium silicate treatment. Trees have been receiving recent spray applications of phosphorous acid to restore tree health, however a row of trees in mid-decline was identified as a treatment site. Nine and ten trees, respectively, were selected for untreated controls or Agsil drench applications (300 mL Agsil per tree, delivered in 20 L of water, i.e., 5000 ppm). Agsil was applied by farm staff in August 2014; February, June, November and December 2015; and May and June 2016. Trees were either pruned heavily or staghorned in March 2015. This trial was visited twice only, prior to treatment application and at the end of the trial in 2016. All trees in the trial were harvested at the time of final tree health assessment (August 2016) and one bin of fruit from each treatment were transported to a commercial packingshed to obtain packout data.

### 4.2. Greenhouse Trials with Mineral Mulch and Mineral Dust

MM and MD are sustainable soil amendment by-products from the manufacture of cement building boards. The major components are sand (approximately 50%), pine pulp, cement and water, and contains 51% total silica (as SiO_2_), with 5% as plant-available silica (monosilic acid) and calcium silicate. The waste boards are milled so that 80% of MM is composed of particles 1000 µm in size, while 88% of MD has particles in the 75–300 µm size range. The effects of Mineral Mulch in avocado have not previously been reported.

Four greenhouse trials with avocado seedlings were undertaken between 2015 and 2017, and consisted of three steps; treatment of avocado seedlings with MD or MM, inoculation of the plants with Ci or Pc and assessment of seedling growth, root necrosis and nutrient uptake.

There were 12 replicate seedlings per treatment, and MD or MM was applied twice, approximately 4–6 weeks apart. Treatments are outlined in result Table 8, Table 9, Table 10 and Table 11 The dust/mulch was gently incorporated into the top 1–2 cm of potting mix. Approximately 30 days later, all plants were re-potted into 140 mm diam. round pots and inoculated with Pc or Ci. Pathogen colonized grain, 10–20 mL (or uncolonized grain as controls) was sprinkled on a thin layer of potting mix sitting at the bottom of each pot. The seedling was placed on top of the inoculum, and the pot back-filled with potting mix. Pc inoculated pots were watered until damp, placed on saucers to simulate waterlogging for 5 days before saucers were removed and all plants watered as required.

Plant height was measured from the base of the stem to the apex and recoded just after inoculation and at the conclusion of the experiment. At the conclusion of the trial, plants were uprooted, roots were washed to remove potting mix, and percentage of necrotic roots was assessed for each plant. Above-ground parts (leaves and stems excised above the seed) and root systems of each plant were placed into paper bags, weighed and dried for three days at 55 °C. Dried roots and leaves from selected treatments of Experiments 1 and 2 were ground to a fine powder using a domestic coffee and spice grinder, then sent to the Analytical Services Unit, School of Agriculture and Food Science, University of Queensland. The samples were prepared by closed-vessel microwave assisted acid digestion, (to solubilize elements in the presence of organic molecules), and then analyzed by ICP-OES (inductively coupled plasma optical emission spectrometry).

### 4.3. Field Trials with Mineral Mulch

Site 1 (Kumbia, QLD): Trees in this orchard were approximately 5 years old at the start of the trial. A poorer section of the orchard was selected, where trees had not established well, or had died. Eight pairs of trees which were stunted, yellow and not thriving were tagged.

Each tagged tree was rated for canopy health (0 = completely healthy and 10 = dead), and height and canopy diameter measured. MM was spread under one of the trees in each pair, in an area corresponding to the canopy drip zone, at the rate of 255 g MM/m^2^. Tree height varied considerably throughout the trial, so MM applications ranged from a minimum of 0.5 kg applied to the smallest tree up to 2.7 kg applied to the largest tree.

The same canopy heath, height and diameter measurements were recorded approximately 10 months after the MM application. Leaves and fruit were collected for quality and nutrient analyses, as described previously, but many of the trees had either no fruit or very little fruit, potentially skewing results.

Site 2 (Goodwood, QLD): Trees in this orchard were approximately 11 years old at the start of the trial, and sections of the orchard are showing fairly uniform decline due to Phytophthora root rot. Ten trees in mid-decline were selected and tagged. MM was applied by manual broadcast to the drip zone of 10 trees in the centre row at the rate of 4.5 kg/tree. Canopy heath was again assessed approximately 10 months later, and leaves and fruit were collected for quality and nutrient analyses.

Site 3 (Childers, QLD): A section of 5-year-old trees near a farm dam was selected, as trees were showing decline due to Phytophthora root rot. Trees were also showing signs of moderate salt (chloride) leaf burn. Canopy health was recorded, and MM applied by manual broadcast to the drip zone of 10 trees in the centre row at the rate of 1.9 kg/tree. Canopy heath was again assessed approximately 10 months later, and leaves and fruit were collected for quality and nutrient analyses, as described previously. There were limited numbers of fruit on trees.

## Figures and Tables

**Table 1:** Tree health and yield data for 2014–2016 from Agsil amendment trial—Site 1, Childers.

	Tree Health ^a^				Yield (kg/tree)
	April 2014	December 2014	March 2015	July 2016	May 2015
No Agsil Drench	5.36	5.81	6.18	4.55	79.9
Agsil Drench	5.39	5.34	5.52	4.74	68.1
*p*	NS	NS	NS	NS	NS

^a^ Tree health is rated on a scale where 0 = healthy and 10 = dead [29]; *n* = 11 trees for No Agsil Drench, *n* = 23 trees for Agsil Drench.

**Table 2:** Packout data collected May 2015 from Agsil amendment trial—Site 1, Childers.

	% Packout by Fruit Grade			
	Premium	2nd	3rd	Total
No Agsil Drench ^a^	50.3	21.1	26.2	97.6
Agsil Drench ^b^	51.3	36.5	10.8	98.6

^a^ No Agsil drench—downgrades due to limb rub (70%), sunburn (20%), insect chew (5%) and pepper spot (5%); ^b^ Agsil drench—downgrades due to limb rub (45%), hail damage (35%), sunburn (20%) and insect chew (5%).

**Table 3:** Tree health data for 2014–2016 from Agsil amendment trial—Site 3, Beechmont.

Treatment	Tree Health ^a^						
	May 2014	December 2014	May 2015	July 2015	December 2015	May 2016	August 2016
Untreated	5.36	5.55	5.09	5.18	4.91	4.82	5.09
Agsil drench	5.36	5.45	5.00	5.55	4.73	4.91	4.64
Agsil spray	5.42	5.92	5.17	4.83	4.17	4.08	4.67
Agsil inject	5.45	5.45	5.27	5.27	5.40	5.09	5.00
*p*	NS	NS	NS	NS	NS	NS	NS

^a^ Tree health is rated on a scale where 0 = healthy and 10 = dead [29]; *n* = 11 for Untreated, Agsil drench and Agsil inject, *n* = 12 for Agsil spray.

**Table 4:** Postharvest quality data collected from fruit harvested in August 2015 from Agsil amendment trial—Site 3, Beechmont.

Treatment	Anthracnose Severity (%)	Stem End Rot Severity (%)		Anthracnose Incidence (%)	Stem End Rot Incidence (%)	Marketability ^a^ (%)
Untreated	9.60	6.97	abc	55.9	19.1	51.4
Agsil drench	9.04	3.65	c	45.5	11.4	58.6
Agsil spray	9.03	5.09	bc	41.7	16.3	58.8
Agsil inject	9.79	9.16	a	48.6	20.9	50.5
*p*	NS	<0.05		NS	NS	NS

Means followed by the same letter are not significantly (*p* < 0.05) different; *n* = 11 for Untreated, Agsil drench and Agsil inject, *n* = 12 for Agsil spray. ^a^ Fruit marketability = less than 5% severity of anthracnose and no stem end rot.

**Table 5:** Postharvest quality data collected from fruit harvested in August 2016 from Agsil amendment trial—Site 3, Beechmont.

Treatment	Anthracnose Severity (%)	Stem End Rot Severity (%)	Anthracnose Incidence (%)	Stem End Rot Incidence (%)	Marketability ^a^ (%)
Untreated	4.77	12.8	25.4	33.0	56.5
Agsil drench	5.34	9.48	27.8	26.8	61.7
Agsil spray	5.72	9.61	27.6	26.3	64.0
Agsil inject	3.79	8.61	20.6	26.3	63.2
*p*	NS	NS	NS	NS	NS

*n* = 11 for Untreated, Agsil drench and Agsil inject, *n* = 12 for Agsil spray. ^a^ Fruit marketability = less than 5% severity of anthracnose and no stem end rot.

**Table 6:** Tree health data for 2014 and 2016 and estimated yield 2016 from Agsil amendment trial—Site 4, Comboyne.

Treatment	Tree Health ^a^		Est. Yield kg/tree
	June 2014	August 2016	August 2016
Untreated	5.3	5.6	47.8
Agsil drench	5.2	4.3	55.0
*p*	NS	NS	ND

^a^ Tree health is rated on a scale where 0 = healthy and 10 = dead [29]; *n* = 9 untreated, *n* = 10 Agsil drench. ND = not determined. Fruit from trees in each treatment was picked into separate bins, and individual yields per tree were not recorded.

**Table 7:** Packout data collected August 2016 from Agsil amendment trial—Site 4, Comboyne.

Treatment	% Packout by Fruit Grade			
	Premium	2nd	3rd	Processing ^a^
Untreated ^b^	56.5	27.8	13.2	2.5
Agsil Drench ^c^	55.6	32.6	9.4	2.3

^a^ Processing = defect; ^b^ Untreated—downgrades due to caterpillar damage (16%), hail (13%), wind rub (19%), sunburn (22%), pepper spot (8%) and anthracnose (3%); ^c^ Agsil drench—downgrades due to caterpillar damage (18%), hail (21%), wind rub (22%), sunburn (24%), and pepper spot (4%). No anthracnose damage.

**Table 8:** Seedling growth, fresh and dry weight of avocado leaves and stems and roots and percent of necrotic roots after amendment of potting mix with Mineral Dust (MD) and inoculation with *Calonectria ilicicola* (Ci), Experiment 1.

Amendment to Potting Media ^a^	Seedling Growth (cm)	Leaves and Stem Biomass ^b^ (g)		Root Biomass (g)		% Necrotic Roots
		FW	DW	FW	DW	
MD (2 g) + Ci	1.37 b	14.9 b	5.92 b	7.25 b	0.91 b	88.2 a
MD (2 g)	10.70 a	28.5 a	8.75 a	17.0 a	2.00 a	12.5 c
MD(6 g) + Ci	0.98 b	13.7 b	4.99 b	4.21 b	0.52 b	94.4 a
MD (6 g)	11.00 a	29.3 a	8.98 a	17.5 a	2.11 a	24.6 b
Control + Ci	1.15 b	16.0 b	5.82 b	6.52 b	0.78 b	87.7 a
Control	10.50 a	26.3 a	8.20 a	15.2 a	1.72 a	17.5 bc
*p*	<0.001	<0.001	<0.001	<0.001	<0.001	<0.001

Mean values followed by the same latter are not significantly different at the *p* value indicated (*n* = 12). ^a^ Two separate doses of MD were applied. First application was 30 days before Ci inoculation and second application three days after inoculation. Control received no MD. ^b^ FW, Fresh weight; DW, Dry weight.

**Table 9:** Seedling growth, fresh and dry weight of avocado leaves and stems and roots and percent of necrotic roots after amendment of potting mix with Mineral Dust (MD) or Mineral Mulch (MM) and inoculation with *Calonectria ilicicola* (Ci), Experiment 2.

Amendment to Potting Media ^a^	Seedling Growth (cm)	Leaves and Stem Biomass ^b^ (g)		Root Biomass (g)		% Necrotic Roots
		FW	DW	FW	DW	
MD (2 g) + Ci	5.67 cd	32.6 bcd	10.8	16.6 b	2.01 abc	41.7 b
MD (2 g)	10.50 a	41.2 a	13.5	27.4 a	2.63 a	0.64 c
MM (2 g) + Ci	3.79 d	28.9 cd	10.0	15.4 b	1.82 bc	47.1 b
MM (2 g)	9.33 ab	38.0 ab	11.7	27.2 a	2.57 ab	1.08 c
Control + Ci	3.13 d	25.8 d	8.78	12.9 b	1.54 c	68.3 a
Control	7.25 bc	35.9 abc	11.3	26.1 a	2.55 ab	1.10 c
*p*	<0.001	<0.01	NS	<0.001	<0.05	<0.001

Mean values followed by the same letter are not significantly different at the *p* values indicated (*n* = 12). ^a^ Two separate doses of MD/MM were applied. First application was 30 days before Ci inoculation and second application three days after inoculation. Control received neither MD nor MM. ^b^ FW, Fresh weight; DW, Dry weight.

**Table 10:** Plant growth and severity of root rot after amendment of potting mix with either Mineral Dust (MD) or Mineral Mulch (MM) and subsequent inoculation with Ci and Pc, Experiment 3.

Amendment to Potting Media ^a^	Seedling Growth (cm)	Leaf and Stem Biomass ^b^ (g)		Root Biomass (g)		% Necrotic Roots
		FW	DW	FW	DW	
MD (2 g)	5.97 a	28.7 ab	9.31 ab	27.3 a	2.73 ab	1.25 c
MD (2 g) + Ci	1.54 b	25.9 ab	9.27 ab	19.3 b	2.11 b	27.1 b
MD (2 g) + Pc	0.83 b	19.9 b	6.78 b	7.98 c	0.73 c	99.2 a
MM (2 g)	6.13 a	33.1 a	10.9 a	28.7 a	3.03 a	0.50 c
MM (2 g) + Ci	1.29 b	26.5 ab	9.59 ab	21.9 ab	2.45 ab	29.6 b
MM (2 g) + Pc	0.79 b	21.0 b	7.35 ab	8.00 c	0.94 c	99.1 a
Control	4.92 ab	27.3 ab	8.74 ab	24.1 ab	2.72 ab	0.92 c
Control + Ci	2.29 ab	24.0 ab	8.18 ab	18.6 b	1.97 b	35.4 b
Control + Pc	0.79 b	21.4 b	7.44 ab	8.83 c	0.86 c	99.3 a
*p*	<0.001	<0.01	<0.05	<0.001	<0.001	<0.001

Mean values followed by the same letter are not significantly different at the *p* values indicated (*n* = 12). ^a^ Two separate doses of MD/MM were applied. First application was 30 days before Ci/Pc inoculation and second application three days after inoculation. Control received neither MD nor MM. ^b^ FW, Fresh weight; DW, Dry weight.

**Table 11:** Fruit quality and canopy health of avocado 10 months after Mineral Mulch application to trees in three orchards in Queensland.

Site and Treatment	*n*	No. Days to Ripe	Anthracnose Severity (%)	Stem End Rot Severity (%)	*n*	Canopy Health 2016	Canopy Health 2017
Kumbia Untreated	16	14.2	4.06 b	14.1 ab	8	4.13 d	3.5 b
Kumbia Mineral Mulch	16	12.6	35.9 a	25.3 a	8	4.25 cd	3.6 b
Goodwood Untreated	15	13.2	3.00 b	1.67 b	20	4.55 bcd	4.6 a
Goodwood Mineral Mulch	12	13.3	1.25 b	17.1 a	10	5.20 a	4.6 a
Childers Untreated	31	12.6	22.9 a	2.74 b	29	4.75 abc	4.5 a
Childers Mineral Mulch	18	11.7	26.4 a	1.67 b	10	4.90 ab	4.4 a
*p*		NS	<0.001	<0.001		<0.01	<0.01

Mean values followed by the same letter are not significantly different at *p* values indicated.
